# Psychological meanings of access to guidance on family relationships in prenatal consultations of a public primary health care service in the context of the COVID-19 pandemic: a clinical-qualitative study on reports of pregnant adolescents in Brazil

**DOI:** 10.1192/j.eurpsy.2023.1100

**Published:** 2023-07-19

**Authors:** E. R. Turato, P. E. Ortolan, R. A. Bastos, M.-P. P. Lipi, M. S. Borges, D. B. Vale

**Affiliations:** Laboratory of Clinical-Qualitative Research, State University of Campinas, Campinas, Brazil

## Abstract

**Introduction:**

In prenatal clinical consultations, what do adolescents talk about, in addition to physical and affective conditions and acquiring information about the general state of the evolution of pregnancy? The symbolic psychological elements that emerge during consultations are important for the handling of family guidelines with the clinical team.

**Objectives:**

To interpret emotional meanings attributed by pregnant adolescents, with the possibility of accessing public health care service in a Brazilian metropolitan city, about talking and listening about Family relationships with the clinical team in prenatal consultations.

**Methods:**

Clinical-qualitative design by Turato. Semi-directed interviews with open-ended questions in-depth conducted online during the pandemic. Sample closed by theoretical information saturation according to Fontanella. Interview material, fully transcribed, was treated by Clinical-Qualitative Content Analysis of Faria-Schützer, with Balintian psychodynamic concepts from Medical Psychology to generate categories of discussion after free-floating readings. Findings were validated by peers from the Laboratory of Clinical-Qualitative Research, at the State University of Campinas.

**Results:**

The sample was closed with 10 pregnant adolescents. Three categories emerged from the analysis: (1) emotional meanings of the non-use the access to health service as a listening space: affective obstacles and social shame; (2) the relationship of complicity with the maternal figure in “competition” with a possible broad psychological relationship with the clinical team; (3) recurrence of teenage pregnancy in the family as a possible obstacle.

**Conclusions:**

The finding so far that the adolescent’s personal reference is reported as the mother figure is also accentuated because the affective relationship with the doctor figure is more fragile. The bond of adolescents is established with the health institution and not with the reference health team.

There is a mismatch between the psychic maturation, still evolving, to the adult identity and the demands of social roles of the pregnant teenager already demanded as an adult. The teenager captures it, and the medical consultation becomes an act that occurs by inertial force. There is a perception of access to the health service and not access to the doctor as someone qualified for the adolescent to talk about relevant personal matters.

**Disclosure of Interest:**

None Declared

